# Wearing individualized 3D printed oral stent to protect normal tissues in patients with nasopharyngeal carcinoma during radiotherapy

**DOI:** 10.1002/acm2.14145

**Published:** 2023-09-07

**Authors:** Wanfu Yang, Zhihua Yang, Wenyan Pan, Hailiang Li, Ting Zhao, Yin Meng, Guofeng Ma, Hongqiang Ye, Jun Shang

**Affiliations:** ^1^ Department of Radiation Oncology General Hospital of Ningxia Medical University Yinchuan Ningxia China

**Keywords:** 3D printing, adverse reaction, nasopharyngeal carcinoma, oral stent, radiotherapy

## Abstract

**Purpose:**

To demonstrate a new individualized 3D printed oral stent in radiotherapy of nasopharyngeal carcinoma (NPC) patients and carry out a comparative analysis combining with clinical case.

**Material and Methods:**

Thirty NPC patients treated in our institution from September 2021 to October 2022 were prospectively enrolled. An individualized 3D printed oral stent was designed for each patient, and one set of computed tomography (CT) slices were obtained with /without wearing the oral stent, respectively. After delineation of target volumes and organs at risk (OARs) on the two CT slices, we finished two treatment plans by using the same target objectives, critical constraints and plan setup for each patient. Finally, the dose distribution and other dosimetric parameters of target volumes and OARs between the two plans were compared.

**Results:**

Tongue volume and tongue length outside of mouth was 10.4 ± 2.5 cm^3^ and 2.8 ± 0.6 cm, respectively, distance between dorsal surface of oral tongue and plate increased from 0.3 ± 0.3 cm to 2.2 ± 0.5 cm by wearing the oral stent. For the target volume, there was no significant difference. However, *D*_max_ of tongue, tongue tip and periglottis decreased significantly from 6352.6 ± 259.9 cGy to 5994.9 ± 478.9 cGy, 3499.8 ± 250.6 cGy to 3357.7 ± 158.0 cGy and 6345.5 ± 171.0 cGy to 6133.4 ± 263.3 cGy, respectively (*p* = 0.000); *D*_mean_ of tongue, tongue tip and periglottis decreased significantly from 3714.7 ± 204.2 cGy to 3169.7 ± 200.9 cGy, 3060.8 ± 216.2 cGy to 2509.6 ± 196.7 cGy and 3853.3 ± 224.9 cGy to 3079.3 ± 222.0 cGy, respectively (*p* = 0.000).

**Conclusion:**

The individualized 3D printed oral stent can reduce the dose of oral tissues and organs, so as to reduce the oral adverse reactions and improve the compliance of patients and the quality of their life. The technique can be used in radiotherapy of NPC patients.

## 1 INTRODUCTION

Nasopharyngeal carcinoma (NPC) is a high radiosensitive malignant tumor and prevails in East and Southeast Asia, particularly Southern China.^1^, ^2^ At present, radiotherapy is regarded as one of the most important treatments for NPC patients. With the emergence and fast development of intensity‐modulated radiation therapy (IMRT), we now can get higher prescription dose and better dose conformity to the target volume, and the 5‐year survival rate has reached over 80%.^3^, ^4^ This improvement of survival rate makes us pay more and more attention to the quality of life of patients after treatment, and therefore pay more attention to the protection of normal tissues during radiotherapy.^5^ However, because the anatomic position of the target volume is close to the oral tissues and nearby organs (especially for those NPC patients with late staging), radiation inevitably causes damage to normal tissues and organs such as oral mucosa, salivary gland and mandible, resulting in complications such as oral mucositis, xerostomia and taste dysfunction.^6^, ^7^, ^8^ These radiation toxicities can aggravate the clinical symptoms of patients, affect their sleep and nutrition intake, and thus adversely affect the effect of radiotherapy and the quality of life after radiotherapy.^9^, ^10^, ^11^

In recent years, many literatures have studied the mechanism, prevention and nursing intervention of oral toxicity in patients with head and neck tumors during radiotherapy.^12^, ^13^, ^14^, ^15^, ^16^, ^17^ Wearing oral stent during radiotherapy is an important measure to reduce oral tissues radiation dose to reduce oral toxicity.^18^, ^19^, ^20^, ^21^, ^22^, ^23^, ^24^, ^25^ When wearing an oral stent, the patient's mouth opens to separate the upper and lower jaw, and the tongue and part of the oral mucosa can be fixed as far away from the high dose area as possible, so as to reduce the oral dose and the occurrence of adverse reactions such as oral mucositis and taste dysfunction. However, the literature reports are mainly on malignant tumors in the oral cavity (OC), nasal cavity and paranasal sinuses, there are few reports on the wearing of oral devices in patients with NPC during radiotherapy.

In the present study, an individualized 3D printed oral stent was designed and applied to the radiotherapy positioning of patients with NPC, and the dosimetric differences of target volume and oral tissues and surrounding organs with and without wearing individualized oral stent were compared.

## 2 MATERIALS AND METHODS

### 2.1 Patients

A total of thirty patients diagnosed with NPC underwent radiotherapy from September 2021 to October 2022 in our hospital were enrolled in this study. Among these patients, 21 were male and 9 were female, and the age of them ranged from 35 to 68 years old (the median age was 53 years old), the Karnofsky Performance Status (KPS) score was 80 or more. Radiation therapy (RT) concurrently with chemotherapy and target therapy (weekly nimotuzumab and cisplatin every 3 weeks) were given to all patients.

#### 2.1.1 The inclusion criteria

All patients underwent necessary imaging examinations and were definitely diagnosed by pathological examination, the imaging examinations included head and neck enhanced CT and MRI, chest CT, abdominal B ultrasound or CT, whole body bone scan and PET‐CT examination if necessary to determine the clinical stage. Patients were required to have no previous history of radiotherapy for head and neck tumors, and no other contraindications to radiotherapy and chemotherapy. This study was approved by the ethics committee of our hospital, and the informed consent form was signed by the patients and their families.

#### 2.1.2 The exclusion criteria

Patients who have previous history of radiotherapy and chemotherapy or complicated with other malignant tumors were excluded. Patients with distant metastasis, halfway to radiotherapy or oral diseases such as oral mucositis, oral ulcer, dry mouth, taste disturbance, dental caries, gingival inflammation and other oral diseases before treatment were also excluded from the study. In addition, patients with a recent history of medication (such as furosemide) leading to adverse reactions such as xerostomia were also excluded.

### 2.2 Designing and manufacture of individualized 3D printed oral stent

#### 2.2.1 Oral preparation

Inform patients and their families of the necessity and process of making 3D printed oral stents, and the informed consent was signed. Oral health education was conducted for the patients, and the correct method of brushing and flossing was instructed. A comprehensive oral examination was conducted, and the whole mouth condition was photographed and retained.

#### 2.2.2 Mold taking

Alginate was used to take the whole oral imprint film, and the intraoral gypsum model was made with anhydrite, and the jaw position relationship was recorded with silicone rubber. During the mold taking process, the patient kept the mouth open, and the distance between the incisors was about 2.5 cm. The tongue body stretched out of the mouth to the maximum extent, and the length between the tip of the tongue and the incisors was recorded, and an indication sign was designed.

#### 2.2.3 Fluoride coating

Clean and blow‐dry the whole tooth surface first, then apply 3 M Clinpro white varnish. Patients are advised to not eat until 30 min later, do not brush teeth or eat overheated or alcoholic food that night.

#### 2.2.4 Oral stent printing

The patient's oral plaster model and jaw relationship were transferred to the jaw frame, and the model was imported into Creamill Mind software by using a digital scanner. The 3D printed oral stent was designed and printed with Objet30 Orthodesk 3D printer. After printing, the 3D printed oral stent was modified and polished appropriately.

#### 2.2.5 Try on the oral stent

**FIGURE 1:**
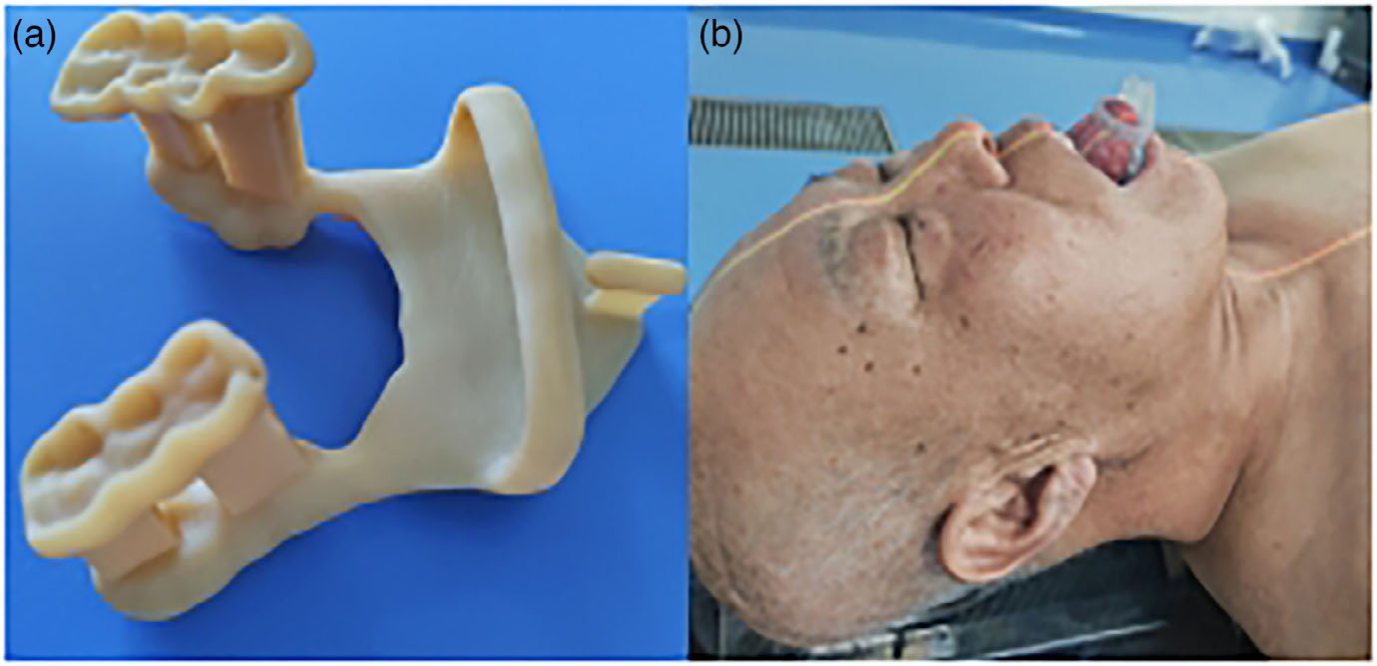
The oral stent (a) and a picture of the patient wearing an oral stent (b).

Comprehensively inspect the patient's mouth, guide the patient to wear and remove the 3D printed oral stent, ensure that the stent is in the correct position, and inform the patient of precautions when wearing the oral stent. Figure 1 shows a finished oral stent and a picture of the patient wearing the oral stent. The oral stent does not touch the patient's oral mucosa when wearing, so there is no pain or discomfort, which enables the patient to maintain the same position of the mouth and tongue for more than 10 minutes to complete the treatment.

### 2.3 CT‐scan, delineation of target volumes and OARs, and treatment planning

All the patients were simulated and immobilized with a thermoplastic mask, lying on the couch, placing hands on the sides of body. For each patient, one set of computed tomography (CT) slices (3 mm) were obtained with/without wearing an individualized 3D printed oral stent respectively from a large aperture CT scanner (Siemens SOMATOM Sensation Open), the scanning range is from the top of the skull to 5 cm below the clavicle. When wearing an individualized 3D printed oral stent, the patient was required to hold the tongue as far out of the mouth as possible so that the tip of the tongue is in the designated position of the stent (Figure 1b). Target volumes and organs at risk (OARs) were delineated on both the two sets of CT slices, and treatment plans were also finished on both the two sets of CT slices respectively to compare the dosimetric differences of the target volume and OARs between wearing and not wearing an oral stent. The gross tumor volume of nasopharynx and positive nodes (GTVp, GTVn) were determined with enhanced CT and MRI fusion images and clinical examination, receiving 69.96 Gy. 60.06 Gy to the high‐risk clinical target volume (CTV1) and 50.96 Gy to the low‐risk clinical target volume and lymphatic drainage region of the neck (CTV2) were prescribed. All targets were treated once daily, 5 times a week in 33 fractions. All the delineations of target volumes and OARs were finished on Pinnacle treatment planning system v. 9.8(Philips Medical System, Milpitas, CA, USA) by an experienced physician and examined and approved by at least one senior physician. Treatment plans were completed using the Accuray treatment planning system which uses a Convolution/Superposition dose calculation algorithm similar to the method described by Ahnesjö A originally in 1989 (Precision, Accuray, Sunnyvale, USA),^26^ and the same target objectives, critical constraints and plan setup were used to complete both plans (with/without the oral stent) of each patient, the accelerator used for treatment was Accuray Radixact X7 (Accuray, Sunnyvale, USA) and the photon energy was 6 MV. After comparison of the two plans of each patient, we chose the treatment plan of wearing an oral stent as the final delivered plan because of its dosimetric advantage, and the patients wore the individualized 3D printed oral stent during each radiotherapy. Onboard MV CT scan validation was performed daily before treatment to assess for changes in anatomy and ensure that patient positioning errors were within allowable limits for each treatment.

### 2.4 Plan evaluation

After completing the optimization and final dose calculation of the plans, we reviewed the two plans (wearing and not wearing an oral stent) of each patient, paying close attention to the differences of dose to target volumes and OARs. In order to get a quantitative analytical result, we reviewed the dose‐volume histogram (DVH) to determine the approximate maximum dose *D*_2%_, approximate minimum dose *D*_98%_, average dose *D*_mean_, conformal index (CI=(Vt,ref∗Vt,ref)/(Vref∗Vt), where Vt refers to the volume of target, Vt,ref refers to the volume of target covered by the isodose line of prescription dose, Vref refers to the volume covered by isodose line of prescription dose) and homogeneity index ($HI=\left(D_{2\%}-D_{98\%}\right)/D_{50\%}$, where $D_{50\%}$ is the median absorbed dose of the target, $D_{2\%}$ and $D_{98\%}$ represent the dose received by 2% and 98% of the target volume) of the targets of the two plans.^27^, ^28^, ^29^ Meanwhile, in order to investigate the dosimetric effect of wearing an oral stent on oral tissues and nearby organs, we compared the maximum dose and mean dose of tongue, tongue tip, periglottis, OC and mandible; the maximum dose, *D*_mean_ and *V*_30Gy_ of parotid of the two plans for each patient. Patient‐specific quality assurance (QA) for all the treatment plans were performed using ArcCheck 2 (Sun Nuclear Corporation, Melbourne, Australia), the QA results were evaluated with gamma analysis method using 3% as the dose difference and 3 mm as the distance to the agreement with a 10% threshold, the percentage‐pass value should be ≥95%.

### 2.5 Statistical method

Statistical analyses were performed using software package SPSS (version 20.0, IBM Inc.), the Paired Sample T‐Test was used to assess the dosimetric differences of target volumes and OARs. The results were expressed by$\bar{x}\pm s$. A probability value of less than 0.05 was considered statistically significant for all analyses.

## 3 RESULTS

### 3.1 Patient characteristics

A total of thirty patients were enrolled into the study. Patient characteristics included age, gender, smoking history, T‐stage, N‐stage and Clinical stage (shown in Table 1).

**TABLE 1:** Characteristics of the study population.

Characteristics	All study patients (*n* = 30)
Age	
Median	53
range	35−68
Gender	
Male	21
Female	9
Smoking history	
No	14
<10 cigarettes a day	9
≥10 cigarettes a day	7
T‐stage	
T2	5(16.7%)
T3	9(30%)
T4	16(53.3%)
N‐stage	
N1	1(3.3%)
N2	16(53.3%)
N3	13(43.3%)
Clinical stage	
III	10(33.3%)
IV	20(66.7%)

### 3.2 Geometrical changes in tongue and oral cavity shape with/without wearing an oral stent

Both the volumes and geometrical shapes of OC changed significantly by wearing an individualized 3D printed oral stent. The mean volume of OC was 257.9 ± 22.6 cm^3^ while wearing an oral stent and 214.0 ± 23.7 cm^3^ without wearing an oral stent (*p* = 0.000). Different OC shapes also led to different distances between the dorsal surface of tongue and the hard palate, the distance between the dorsal surface of tongue and the hard palate was 0.3 ± 0.3 cm without wearing an oral stent, while it was expanded to 2.2 ± 0.5 cm when wearing an individualized oral stent (*p* = 0.000).

**FIGURE 2:**
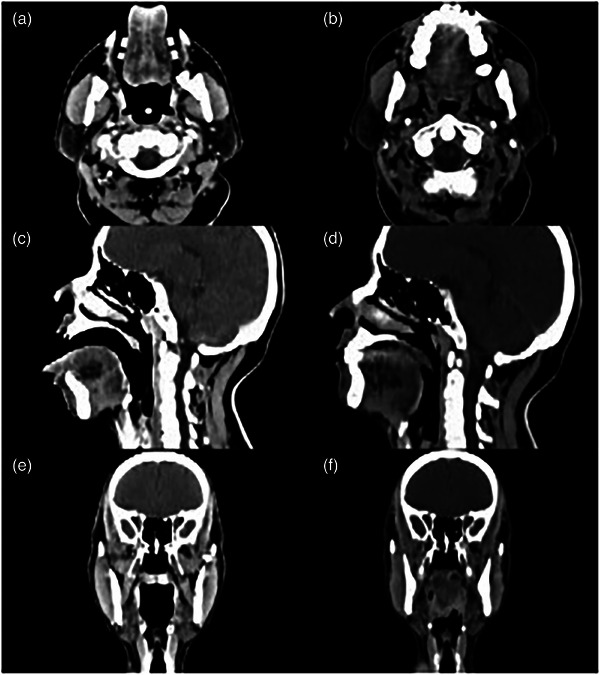
Changes in oral cavity with/without wearing an oral stent (a,c,e = images form CT‐simulation with an oral stent; b,d,f = images form CT‐simulation without an oral stent).

Furthermore, the patient's tongue was elongated to be out of the mouth while wearing an individualized oral stent, the length and volume of tongue outside of mouth from anterior surface of lip was 2.8 ± 0.6 cm and 10.4 ± 2.5 cm^3^, while the entire tongue was inside of mouth without wearing an individualized oral stent. Figure 2 and Table 2 demonstrate geometric changes in OC with/without wearing an individualized oral stent. In order to obtain a quantitative result of the positioning offsets of the tongue during the course of radiotherapy, the onboard CT system was used to get the CT images of the patient for registration before each treatment, and the results were 0.15 ± 0.05 cm in vertical, 0.16 ± 0.04 cm in longitudinal and 0.15 ± 0.04 cm in lateral direction.

**TABLE 2:** Geometric changes in oral cavity and tongue with/without wearing an individualized oral stent.

	Wearing oral stent	Not wearing oral stent	T‐value	*p*‐value
Oral cavity volume	257.9 ± 22.6 cm^3^	214.0 ± 23.7 cm^3^	22.555	0.000
Tongue volume outside of mouth	10.4 ± 2.5 cm^3^	0	22.527	0.000
Tongue length outside of mouth	2.8 ± 0.6 cm	0	25.909	0.000
Dorsal surface of oral tongue to plate	2.2 ± 0.5 cm	0.3 ± 0.3 cm	22.625	0.000

### 3.3 Dosimetric comparison of target volumes and OARs between the two plans of each patient

As mentioned above, after obtaining the two sets of CT slices (with/without wearing an individualized 3D printed oral stent) of each patient, we finished treatment planning with the same plan setup and constraints on both the two CT slices, and made a dosimetric comparison of target volume and OARs. The mean gamma passing rate of all the delivered plans was 99.3%. As shown in Table 3, both plans of each patient could meet the clinical requirements, there was negligible difference in the average dose (*D*_mean_), approximate minimum dose (*D*_98%_) and approximate maximum dose (*D*_2%_) of target volume between the two plans. The conformal indexes (CI) and the homogeneity indexes (HI) changed little, also negligible. The HI of GTV is much smaller than that of PTV, mainly because PTV contains GTV which has a higher prescription dose, so the homogeneity of PTV is worse than that of GTV.

**TABLE 3:** Dosimetric comparison of target volumes between the two plans of each patient.

	Plan (wearing oral stent)	Plan (not wearing oral stent)	T‐value	*p*‐value
PTV				
CI	0.855 ± 0.010	0.856 ± 0.012	−0.232	0.818
HI	0.227 ± 0.013	0.227 ± 0.011	0.242	0.810
*D*_mean_(cGy)	6470.8 ± 62.699	6480.0 ± 75.381	−0.501	0.620
*D*_98%_(cGy)	5995.0 ± 22.947	5987.4 ± 25.838	1.113	0.275
*D*_2%_(cGy)	7384.6 ± 43.236	7391.2 ± 73.868	−0.501	0.620
GTV				
CI	0.804 ± 0.041	0.792 ± 0.084	0.958	0.346
HI	0.065 ± 0.005	0.065 ± 0.008	0	1
*D*_mean_(cGy)	7219.5 ± 116.5	7227.7 ± 70.5	−0.435	0.667
*D*_98%_(cGy)	6975.7 ± 25.2	6978.8 ± 29.1	−0.330	0.744
*D*_2%_(cGy)	7457.0 ± 39.4	7461.2 ± 78.9	−0.321	0.750

**FIGURE 3:**
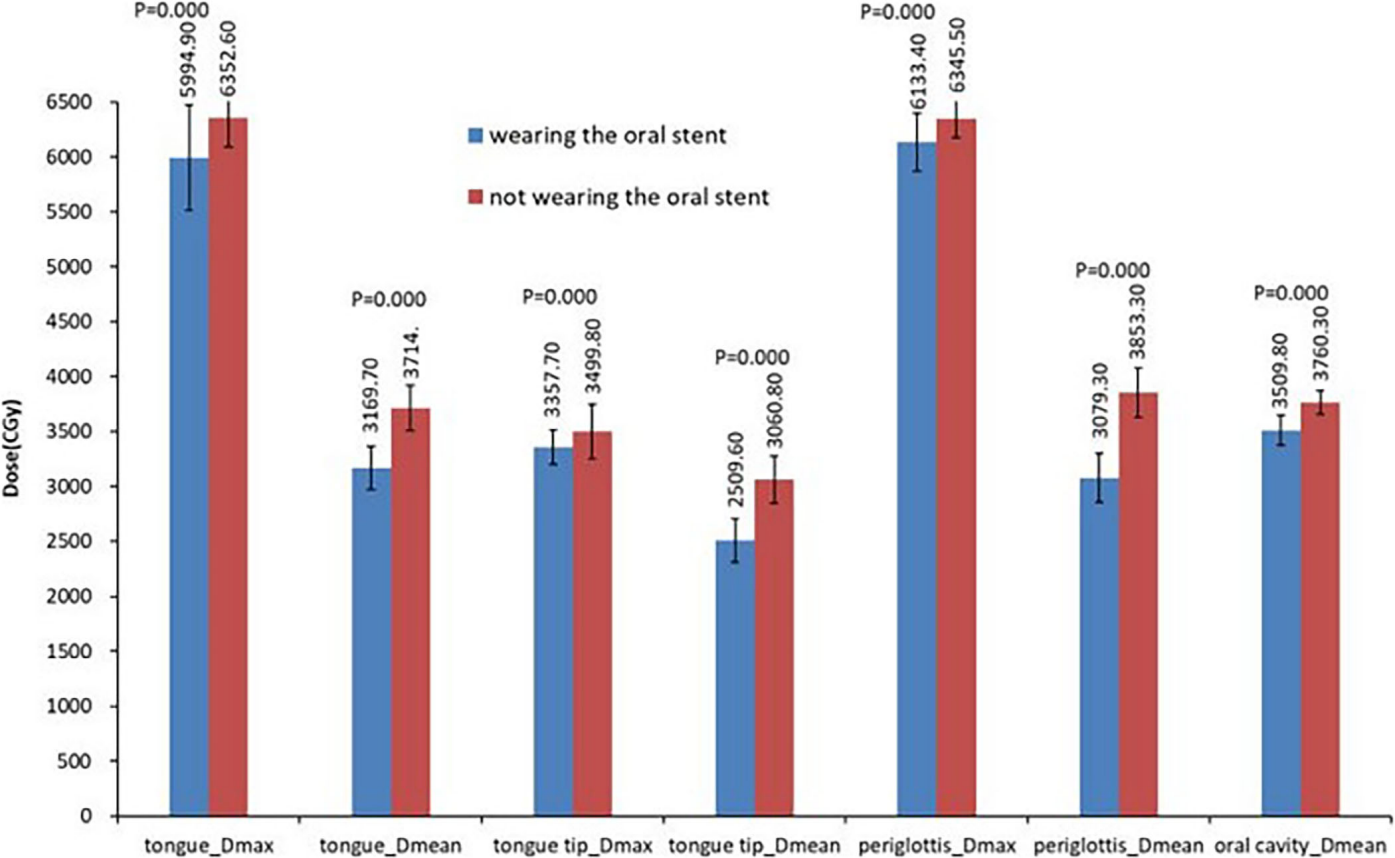
Dosimetric comparison of tongue, tongue tip, periglottis and oral cavity with/without wearing an oral stent.

The statistical results of doses to OARs are presented in Table 4 and Figure 3. The maximum dose (*D*_max_) and mean dose (*D*_mean_) of tongue, tongue tip and periglottis decreased significantly by wearing an oral stent during radiotherapy, the mean dose of OC also decreased significantly from 3760.3 ± 107.1 cGy to 3509.8 ± 138.3 cGy, the *p*‐value was 0, while the change in *D*_max_ of OC were negligible. Additionally, there are almost no changes in *D*_max_ and *D*_mean_ of mandible and parotid between the two plans of each patient, the *V*_30Gy_ of parotid also changed insignificantly.

**TABLE 4:** Dosimetric comparison of OARs between the two plans of each patient.

	not wearing oral stent	wearing oral stent	T ‐value	*p*‐value
Tongue				
*D*_max_(cGy)	6352.6 ± 259.9	5994.9 ± 478.9	−5.488	0.000
*D*_mean_(cGy)	3714.7 ± 204.2	3169.7 ± 200.9	−13.418	0.000
Tongue tip				
*D*_max_(cGy)	3499.8 ± 250.6	3357.7 ± 158.0	−4.913	0.000
*D*_mean_(cGy)	3060.8 ± 216.2	2509.6 ± 196.7	−23.125	0.000
Periglottis				
*D*_max_(cGy)	6345.5 ± 171.0	6133.4 ± 263.3	−5.399	0.000
*D*_mean_(cGy)	3853.3 ± 224.9	3079.3 ± 222.0	−13.208	0.000
Oral cavity				
*D*_max_(cGy)	7216.3 ± 260.4	7247.0 ± 263.5	1.594	0.122
*D*_mean_(cGy)	3760.3 ± 107.1	3509.8 ± 138.3	−14.855	0.000
Mandible				
*D*_max_(cGy)	6965.1 ± 204.5	6968.4 ± 208.6	0.072	0.943
*D*_mean_(cGy)	4007.6 ± 186.0	4035.4 ± 151.7	1.920	0.065
Parotid_L				
*D*_mean_(cGy)	3042.9 ± 87.0	3061.8 ± 76.0	1.644	0.111
*V*_30Gy_(%)	40.6 ± 2.2	40.7 ± 1.6	0.520	0.607
Parotid_R				
*D*_mean_(cGy)	3028.8 ± 55.7	3037.0 ± 74.0	0.606	0.549
*V*_30Gy_(%)	40.5 ± 1.6	40.6 ± 1.4	0.364	0.719

## 4 DISCUSSION

Radiation‐induced oral / oropharyngeal mucositis can occur in almost all patients with NPC during/after radiotherapy. In order to reduce the degree of adverse reactions such as oral mucosal reaction and taste damage, many reports have demonstrated the use of oral stents to reduce the dose of oral tissues.^30^, ^31^, ^32^ When wearing an oral stent during radiotherapy, the patient's mouth opens to separate the upper and lower jaw, which can keep the normal tissues of the mouth as far away from the target volume as possible. In this way, the tongue, floor of the mouth, mandible and their surrounding tissues are effectively away from high dose area, thus reducing the occurrence or symptoms of adverse reactions such as dry mouth, oral mucositis and taste dysfunction.

The design of the oral stent reported in the present study is different from those reported in the previous literature. When wearing the oral stent, the patient opened the mouth and stuck the tongue out of mouth as far as possible. Because of the ingenious design of the oral stent, the tongue body outside the mouth can be fixed and the length of the tongue protruding out of the mouth is the same during and among each treatment, which has the advantage of ensuring the repeatability of the patient's positioning. In addition, wearing the oral stent also makes the tongue body far away from the target area, so as to reduce the radiation dose of the tongue body, thus reducing the reaction of tongue mucosa and taste damage caused by radiotherapy. As can be seen in Table 4, *D*_max_ and *D*_mean_ of tongue, tongue tip and periglottis are significantly reduced. Because the volume of OC, especially the part away from the target area becomes larger after wearing the oral stent, the *D*_mean_ of OC becomes significantly smaller, but because wearing the oral stent does not change the distance between OC and the target area, the *D*_max_ of OC does not change significantly. Meanwhile, as can be seen in Table 3, there is almost no change in the dosimetric parameters of the target volumes.

The basic principle of radiotherapy is to reduce the exposure to normal tissues and organs as much as possible on the premise of ensuring the local control rate of tumor. The dosimetry data of this study showed that the coverage of the target area was not affected when patients wear the individualized oral stent, and there was no significant difference in conformal index and homogeneity index between treatment plans of patients with/without wearing an individualized oral stent. This shows that wearing the individualized oral stent has no dosimetric impact on the target area. However, there was a significant difference in the dose of tongue and OC between wearing and not wearing the oral stent during radiotherapy, mainly because the ingenious design of the oral stent kept the tongue away from the high‐dose area of the nasopharynx during radiotherapy. Meanwhile, the use of this individualized oral stent has no obvious impact on the radiation dose of mandible and parotid gland, the main reason is that parotid gland is located on both sides of the mouth, and the open‐mouth and extend‐tongue position has no obvious influence on its position. In addition, most of the cases in this study are N2 or N3 stage, and the target area of radiotherapy includes at least one side of Ib area, which is adjacent to mandible, so when wearing the oral stent, the dose to the mandible was not significantly changed. Generally speaking, from the point of view of dosimetry, the individualized oral stent did not affect the dose distribution of the target area, but had a protective effect on OARs including tongue and OC, which may effectively reduce the occurrence of adverse reactions such as oral mucositis and taste disturbance in NPC patients.

Radiotherapy to the head and neck region can cause radiation‐induced oral mucositis which may ultimately lead to poor quality of life.^33^, ^34^ In extreme cases, lesions are characterized by the formation of large and painful ulcers, which have an obvious impact on the patient's quality of life, and may to a large extent limit activities such as eating, speaking, and even swallowing saliva.^35^ Sonis proposed a five‐stage model to describe the occurrence and development of oral mucositis: initiation, upregulation and message generation, signaling and amplification, ulceration and healing.^36^, ^37^ The initial clinical symptoms of oral mucositis include mucosal erythema and superficial sloughing, which may occur when the cumulative radiation dose reaches 20−30 Gy after 3 weeks of the beginning of radiotherapy, at which, the intact mucosa begins to rupture, followed by ulceration,^38^, ^39^ ulcers are usually covered with a white fibrinous pseudomembrane. The clinical severity of oral mucositis is closely related to the radiation dose, most patients who receive more than 50 Gy to the oral mucosa develop severe ulcerative oral mucositis,^40^ while the lesions usually resolve within about 2−4 weeks after the last fraction of radiotherapy.^5^, ^41^ In our study, we have preliminarily observed that the degree of oral adverse reactions after wearing the oral stent were relatively mild compared to historical data without wearing an oral stent in NPC patients, the data of oral mucositis and other adverse reactions after the final treatment are being followed up.

Taste injury is also a common early adverse reaction of radiotherapy in patients with head and neck cancers. There is abundant mucosa, taste buds and minor salivary glands on the surface of the tongue, which have important functions such as assisting in chewing and swallowing food, perceiving taste and pronunciation. Sonja Stieb reported on a study of oral stents used in radiotherapy with oropharyngeal cancer patients, it was found that oral stents brought a significant improvement in the proportion of patients with moderate/severe taste impairment (2% vs.15%, *p* = 0.047).^42^ The study of Hideomi Yamashita et al. showed that under the condition that the tip of the tongue was not irradiated, no obvious taste disturbance was found when the base of the tongue was irradiated with a higher dose, but even a *D*_mean_ of the tip of the tongue lower than 20 Gy can cause obvious taste disorders, it was suggested that the dose of the tip of the tongue should be reduced as much as possible.^43^ In the present study, compared to not wearing an oral stent, patients wearing the individualized oral stent during radiotherapy can keep the tongue (especially the tip of the tongue) far away from the target area, thus reducing the radiation dose and severity of taste dysfunction.^44^

In this study, the application of an individualized 3D printed oral stent in reducing oral dose and oral adverse reactions in patients with NPC was discussed in detail, however the observation period for oral reactions was short, we will continue to observe and record the progress and recovery of oral mucosal reactions and taste dysfunctions of the 30 patients in this study. By comparing the long‐term oral adverse reaction and recovery of patients with wearing the individualized oral stent, the advantage of the oral stent will be furtherly discussed. In addition, although the method used in this study reduced the dose to OC and tongue to some extent while ensuring the radiation dose to the target volume, the degree of reduction was still not enough. In future studies, we will try to improve the oral stent on the basis of the current design to reduce the dose to OC and tongue more.

## 5 CONCLUSION

Radiotherapy is commonly used in the treatment of NPC patients, yet because of the close proximity of tumors to critical OARs, the oral toxicity is inevitable. The preliminary results of this study are encouraging, we have found that the utilization of the individualized 3D printed oral stent can reduce the dose to oral tissues and organs, while there was little impact on the dose distribution of target volume, the reduction of dose to OARs can furtherly translate into the reduction of oral adverse reactions, so as to improve the compliance of patients and the quality of their life.

## AUTHOR CONTRIBUTIONS

The authors confirm contribution to the paper as follows: Study conception and design: Zhihua Yang, Wanfu Yang, and Jun Shang. Data collection: Wenyan Pan and Ting Zhao, and Guofeng Ma. Analysis and interpretation of results: Hailiang Li and Hongqiang Ye. Draft manuscript preparation: Zhihua Yang and Yin Meng. All authors reviewed the results and approved the final version of the manuscript.

## CONFLICT OF INTEREST STATEMENT

The authors declare that the research was conducted in the absence of any commercial or financial relationships that could be construed as a potential conflict of interest. All authors revised the content and approved the final manuscript.

## ETHICS STATEMENT

The study was conducted in accordance with the Declaration of Helsinki of 1975, revised in 2013. The protocol was approved by the ethics committee of the General Hospital of Ningxia Medical University (KYLL‐2022‐0426). All individual participants in the study provided written informed consent.

## CONSENT FOR PUBLICATION

Informed consent has been obtained to publish the information/image(s) in an online open‐access publication from all participants.

## Data Availability

The authors declare that all data supporting the findings of this study are available within the article and its supplementary information files. The datasets used and/or analyzed during the current study available from the corresponding author on reasonable request.
